# A study on the integrity and authentication of weather observation data using Identity Based Encryption

**DOI:** 10.1186/s40064-016-2834-9

**Published:** 2016-08-02

**Authors:** Jung Woo Seo, Sang Jin Lee

**Affiliations:** Graduate School of Information Security, Korea University, 145 Anam-ro, Seongbuk-gu, Seoul, Korea

## Abstract

Weather information provides a safe working environment by contributing to the economic activity of the nation, and plays role of the prevention of natural disasters, which can cause large scaled casualties and damage of property. Especially during times of war, weather information plays a more important role than strategy, tactics and information about trends of the enemy. Also, it plays an essential role for the taking off and landing of fighter jet and the sailing of warships. If weather information, which plays a major role in national security and economy, gets misused for cyber terrorism resulting false weather information, it could be a huge threat for national security and the economy. We propose a plan to safely transmit the measured value from meteorological sensors through a meteorological telecommunication network in order to guarantee the confidentiality and integrity of the data despite cyber-attacks. Also, such a plan allows one to produce reliable weather forecasts by performing mutual authentication through authentication devices. To make sure of this, one can apply an Identity Based Signature to ensure the integrity of measured data, and transmit the encrypted weather information with mutual authentication about the authentication devices. There are merits of this research: It is not necessary to manage authentication certificates unlike the Public Key Infrastructure methodology, and it provides a powerful security measure with the capability to be realized in a small scale computing environment, such as the meteorological observation system due to the low burden on managing keys.

## Background

With the advanced information technology, people-oriented communications connecting people to life is changing into a man-to-machine and machine-to-machine environment. Even in regards to weather information services, there has been technical progress: analog methodology to collect information from meteorological observation sensor has changed to machine-to-machine allowing digital communications.

Weather information measured from meteorological observation sensors get transmitted to a gathering server, and is utilized as a database for weather forecasting. If weather information collected from the observation system being forged or falsified, it produces incorrect weather forecasting in cases of dangerous meteorological phenomena, and further, this could cause threats to national security and huge economic losses. Likewise, weather information is utilized to predict economic activity and provide an environment ensuring lives and the safety of people, despite the dangerous meteorological phenomena.

Weather information is an important factor for national security and the economy. It is essential to consider securing of the integrity and confidentiality of observed meteorological data. Confidentiality and integrity are important, for if meteorological data on dangerous meteorological phenomena is forged or falsified by cyber-attack, thus producing incorrect weather information, the results could have severe influence on both national security and the economy.

In order to secure the integrity of collected data from meteorological observation sensors, an Identity Based Signature methodology was employed. Also, with mutual authentication of the data logger and the Local Acquisition Unit (LAU) server, confidentiality of the meteorological observation data and protection from physical attack were secured. There are merits of the Identity Based Signature methodology: there is no necessity for the preliminary distribution of keys, it can be utilized on small electronic devices with low power due to the low amount of calculation and keys with short length. It also provides non-repudiation functions.

This paper consists of seven sections: “Related studies” section discusses related research, “Attack threats for meteorological observation communication networks” section contains information about attack threats on meteorological observation communication networks, “Encryption method for the interchange of meteorological observation data” section includes encryption system designed for the interchange of metrological observation data, “System model” section contains a model of the system, “Implementation and comparative analysis” section is about the implementation and comparative analysis, and “Conclusion” section finishes the thesis with a conclusion.

## Related studies

### Overview of cryptographic operations

In 1984, Shamir suggested first cipher system called IBE (Identity Based Encryption, which was a solution for the weak point of public key systems: managing public keys effectively (Shamir 1984; Bellovin and Merritt 1993; Boneh et al. 2001; Cramer and Shoup 2002; Boldyreva 2003). In other words, unlike existing public key systems using random bit strings as public keys, the IBE uses the same attribute value as the Identity, which does not require additional memory to save public keys (Zhang et al. 2004).

### Bilinear pairings

Allowable bilinear pairings $\hat{e}$ is defined in groups G and F, which have the rank of the same prime number. $G^{*} , \overrightarrow {{Z_{q}^{*} }}$ represents $G\backslash \left\{ O \right\}$ and $Z_{q}{\backslash } \left\{ O \right\}$ (O is identity element of G). *G* and *F* are addition groups and multiplication groups, correspondingly. *G* is implemented with a group of points on an elliptic curve that has the index of a small MOV (Menezes Okamoto Vanstone). *F* is implemented with a multiplication group in a finite field. Attributes of allowable bilinear pairings $\hat{e}:G \times G \to F$ are as follows:

- Bilinear: when $R_{1} , R_{2} \in G$, $a, b \in \overrightarrow {{Z_{q}^{*} }}$, Bilinear Pairings $\hat{e}$ satisfies $\hat{e}\left( {aR_{1} , bR_{2} } \right) = \hat{e}\left( {R_{1} , R_{2} } \right)^{ab}$
- Non-degenerate: $\hat{e}$ does not pair with any element on $\text{G} \times {\text{G}}$ as the identity element of ${\text{F}}$, and if R is a generating element of *G*, then, $\hat{e}\left( {{\text{R}}, {\text{R}}} \right)$ is a generating element of F
- Computable: about ${\text{R}}_{1} , {\text{R}}_{2} \in {\text{G}}$, pairing $\hat{e}\left( {{\text{R}}_{1} ,{\text{R}}_{2} } \right)$ is efficiently computable

### Boneh Franklin encryption algorithm

**Fig. 1:**
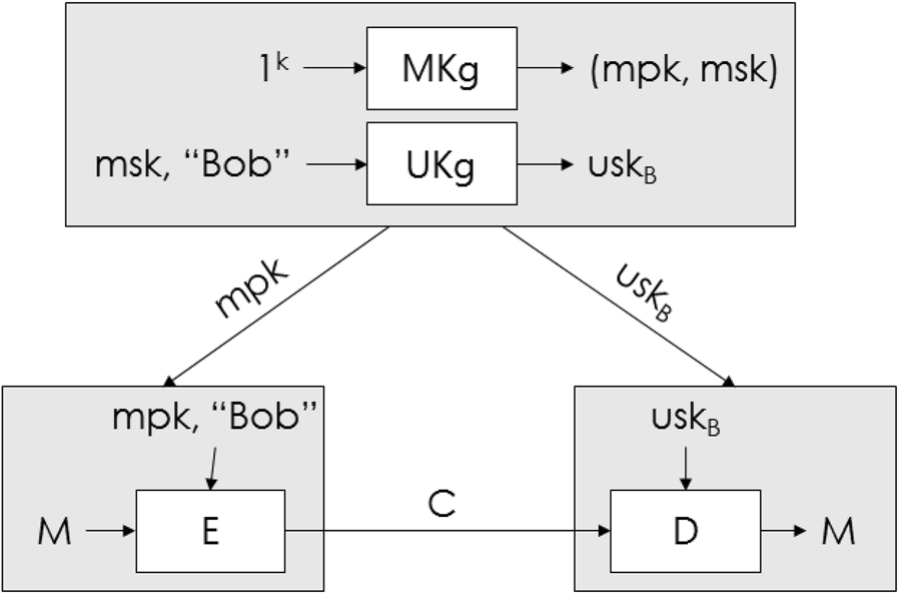
Algorithm for encryption based on ID (Boneh and Boyen 2004; Emerging S&T Report 2005)

An Identity Based Encryption algorithm of Boneh Franklin consists of elements shown in Fig. 1, and the encryption setting is defined as the following:

First, the Private Key Generator (PKG) specify group G generated by $P \in G^{*}$, and Bilinear Pairings $\hat{e}:G \times G \to F$, specify two hash functions $H_{1} :\left\{ {0, 1} \right\}^{*} \to G^{*}$ and $H_{2} :F \to \left\{ {0, 1} \right\}^{l}$ (*l is length of plaintext*.) and the PKG release specification of not only *P*_*PKG*_, but also group G, F and the Hash function $H_{1} , H_{2}$. Second, the receiver Bob receives his private key *D*_*ID*_ = *sQ*_*ID*_ from the PKG. Third, the sender Alice encrypts the message $M \in \left\{ {0, 1} \right\}^{l}$ with Bob’s ID, and computes *U* = *rP* and $V = H_{2} (\hat{e}\left( {Q_{ID} ,P_{PKG} )^{r} } \right) \oplus M$. *r* is a randomly selected value from *Z*_q_^*^, and *Q*_*ID*_ is *Q*_*ID*_ = *H*_1_(*ID*), and ciphertext is $C = \left( {U, V} \right)$. Fourth, after Bob receives the ciphertext, he restores the message *M* with the following computation $M = V \oplus H_{2} \left( {\hat{e}\left( {D_{ID} , U} \right)} \right)$. The safety of the ID based encryption algorithm of Boneh Franklin as far as a chosen plaintext attack was proved in a random oracle model under the hypothesis that it is hard to compute BDH (Bilinear Diffie Hellman) problems with it (Shamir 1984; Cocks 2001; Boneh et al. 2003; Smart 2003; Sahai and Waters 2004; Menezes 2005).

### Signature algorithm of Shamir

**Fig. 2:**
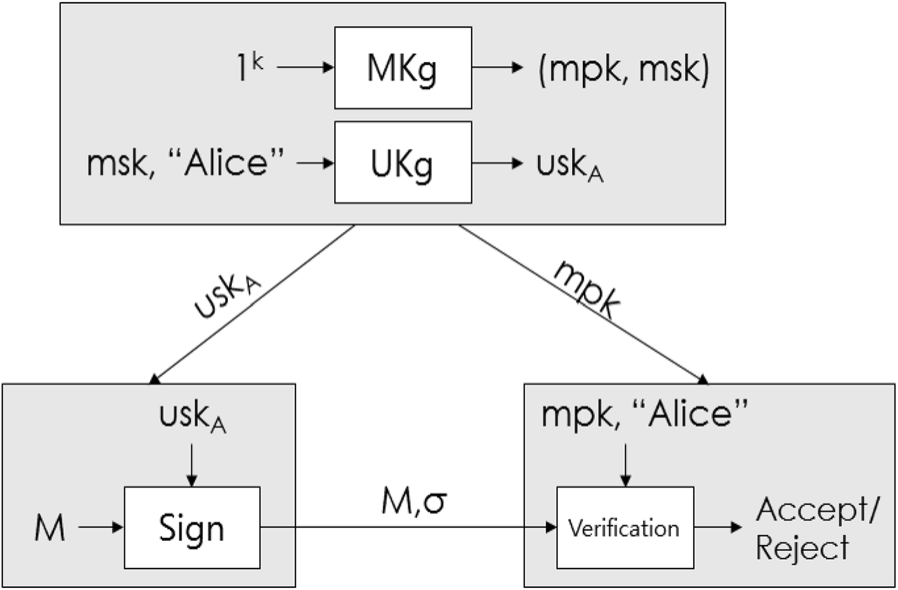
Algorithm for signature based on ID (Boneh and Boyen 2004; Emerging S&T Report 2005)

Shamir’s ID based signature algorithm consistes as shown in Fig. 2, and the setting is defined as the following:

First, the PKG computes ${\text{n}}$ as the multiplication of two big prime numbers in order to choose the system parameter, and chooses a one-way function ${\text{h}}$ after it chooses *ϕ*(*n*) and a big co-prime number ${\text{e}}$ (*ϕ* is Euler’s totient function).

In addition, ${\text{params}}\;\left\langle {n, e, h} \right\rangle$ and master key (a factorized number of *n*) are distributed. Second, in the process of extracting keys, the ID gets sent to the user and the PKG computes the private key *g* corresponding to $g^{\text{e}} = {\text{ID mod n}}$. Third, the user with the private key *g* chooses a random number *r*, and $r^{{h\left( {t,n} \right)}} \,\bmod \, n$ is computed after *t*^*e*^ = *r* mod *n* is computed and a signature $\sigma = \left\langle {s, t} \right\rangle \in Z_{n} \times Z_{n}$ is performed. Fourth, the signature $\sigma = \left\langle {s, t} \right\rangle$ of user with the ID verifies the fact that $s^{e} = ID \cdot t^{{h\left( {t,n} \right)}} \bmod \,n$ is valid. For signatures, the powers of integers, for the verification process, a multiplication of integer and a hash operation, are required. Stability of the signature method is based on the difficulties of the integers factoring problem (Boneh et al. 2001; Paterson 2002; Zhang and Kim 2002; Bellare et al. 2004; Ateniese and Medeiros 2004; Emerging S&T Report 2005; Youngblood 2005; Sarkar and Chatterjee 2006).

### Collection and interchange of meteorological observation data

The AWS (Automatic Weather System) collects data such as current wind speed, wind velocity, air temperature, dew point temperature, and air pressure in order to improve the automation of surface weather observation tasks. The AWS is installed in places with no other meteorological stations to monitor dangerous meteorological phenomena such as severe rainstorms, hail, thunderstorms and wind gusts in real time. It is installed in areas such as islands and mountains, which are not otherwise equipped with monitoring devices, in order to monitor sudden dangerous weather conditions. The collected data is utilized as initial input data for numerical weather prediction models.

### Collection of meteorological observation data

**Fig. 3:**
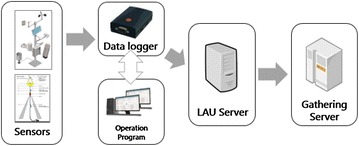
Flowchart on weather observation data

Five factors, wind direction, wind velocity, air temperature, amount of precipitation, and whether or not there is precipitation, are observed automatically, and the collection cycle of observation data is set in minutes. The data logger receives data from sensors and processes it. When the processed data is converted from analog signals into digital signals within the data logger, it transmits the data to the collection and process server through either wired or wireless communication. Figure 3 shows the flow chart of the transmission of observed data from the observation system to the LAU (Local Acquisition Unit) server (AMOFSG 2011; WMO 2014; Benghanem 2009).

Weather information needs to provide precision, speed, proper timing, accessibility, and applicability, and the observation data from the system gets collected every minute in order to create ASCII files. A meteorological observation communication network consists of wired communication utilizing proprietary cables or Internet cables provided by the ISP (Internet Service Provider) and a wireless networking environment based on a M2 M (Machine to Machine) platform. Observation data is transmitted to collection servers using sockets.

## Attack threats for meteorological observation communication networks

### Subjects and causes of the threats

Threats on weather information occur in artificial forms, such as malicious invaders or adversarial countries; physical forms such as a malfunctioning device or natural disasters; and environmental forms. While weather information influences the safety and economic activities of citizens’ day-to-day activities, it also has an influence on the outcomes of war. Since weather information is especially important during wartime situations, there are special organizations in charge of weather forecasting in each country and they are setup to support weather information by government enactment. In South Korea, the Air Force’s 73th Weather Task Force is in charge of weather information, while in the United States, the Air Force’s 557th Weather Wing Unit handles weather information.

Types of external threats on meteorological observation systems or networks can exist in various forms, they can perform physical attacks and produce false weather forecasting with the forgery and falsification of observed data. They have clear attack purposes, and they are created in order to cause threats to national security by destroying related facilities resulting economic and social chaos.

### Security requirements in meteorological observation environment

Since weather information is utilized as a database in order to perform precise forecasting, the construction of a security network environment for providing stable services in cases of dangerous meteorological phenomena and wartime is required. WMO (World Meteorological Organization) provides a “Guide to Information Technology Security” in order to protect the services of meteorological data through the network (WMO 2013).

In the aspect of information security, one must consider confidentiality, integrity, and the availability of metrological data. Integrity is the core factor of weather information security. The reason for this is when weather information is either forged or falsified, it can produce incorrect weather forecasting that could result in casualties and economical damage through unexpected meteorological changes, such as severe rainstorms, storm surges, earthquakes, and volcanic eruptions. A list of things needed to be specifically considered in the aspect of information security of weather information is as follows.

#### Confidentiality

Weather forecasting services contribute to the safety of citizens and national economics and provide services to related organizations and citizens in various forms. Likewise, weather information is substantially related to lives of citizens, while it can also be a core factor into winning a war during wartime. Therefore, due to the fact that securing weather information in a monopolistic way can be a core factor in order to construct the strategy, encrypted communication is required.

#### Integrity

Meteorological observation data (surface observation, marine weather observation, and upper air observation) is to be transmitted to a collection server with a logger or data transmitting server through a wired or wireless network. At the present time, since data transmitting is done in form of unencrypted texts in an end-to-end communication, an attacker could perform attacks by transmitting falsified data values by accessing the gathering server after neutralizing the LAU server with the denial of a service attack.

#### Availability

In order for weather forecasting to be reliable, meteorological observation data needs to be accessible and able to be utilized without delay. It should also be kept in a form in which it does not get lost and yet is always accessible. However, the paralysis of a meteorological observation communication network by DoS (Denial of Service) attack, human error, and physical hijacks or the disabling of the ability to provide normal services by causing the exhaustion of the meteorological observation system could be done at any time. Table 1 shows the comparison of properties on cryptography system.

**Table 1:** Comparison of properties on cryptography system

	PKI	Symmetric	IBE
Key generation	Difficult	Easy	Easy
Key hijack	Difficult	Easy	Difficult
Key distribution	Difficult	Easy	Easy
Key management	Difficult	Easy	Easy
Dev. convenience	Low	High	Middle
Applicability	Low	High	Middle
Performance	Low	High	Middle

## Encryption method for the interchange of meteorological observation data

### Public key infrastructure (PKI)

Since the system is based on a Public Key Infrastructure, it is required to perform management functions, such as the validating of the efficiency of public keys and the discarding of public keys. However, an Identity Based Encryption system utilizes identifiable information. Therefore, it does not require validating of the efficiency of public keys.

In an Identity Based Encryption methodology, the individual creates private keys and then the public keys respond to that which will be created. A certificate authority will certify the public keys. When a private key is exposed, it needs to be discarded and a new key will be generated with the certification process. In order to apply an Identity Based Encryption system for a meteorological observation system, each device needs to be able to generate private keys, public keys, and certification. It also needs to authenticate the public keys. If keys need to be replaced due to private keys of meteorological observation system getting exposed, or due to obstacles, then the procedure of authentication and restoration of an Identity Based Encryption system is complicated (Bellare et al. 1998; Padmavathi and Ranjitha Kumari 2013). As a result, the public key authentication needs to be performed after the generation of public and private keys in an observation sensor.

### Symmetric cryptography

The system needs to manage the encryption keys for the encryption and decryption key for decryption to be the same and make it confidential to anyone other than the sender and receiver. Since the internal structure of the algorithm consists of a combination of a simple substitution and its transpose, an algorithm can be easily developed easy and work quickly in the encryption method of the meteorological observation system. However, because the sender and receiver are required to share the same keys, when information gets shared with multiple systems, difficulty into generating and managing multiple keys may arise.

Additionally, when symmetric cryptography is utilized between the observation sensor and the gathering server, key interchanging problems, due to the replacing of devices during the emergency situation, such as obstacles, may arise, and in cases when the symmetry key is hijacked during wartime or due to an external attacker, it may be used maliciously for things such as falsifying the meteorological observation data (Padmavathi and Ranjitha Kumari 2013; Joux 2000).

### Identity based encryption

This system is a type of public key algorithm, and it has the advantage that it does not require validating the efficiency of the public keys due to the fact that it utilizes specific information already disclosed. Since the key managing system performs the creating key stage for each user and the system knows the private keys, it might not be appropriate for a public Internet network; however, it can be used effectively in an independent network environment for the aspect of security.

As a result, an administrator knows the information about the private keys of an observation system in Identity Based Encryption system, and the validating procedure for the public keys is not required. Therefore, in an emergency situation, replacing the private keys or restoring the system, it does not require the validating procedure, thus making the creating of new private keys in the key management center and restoration of the system parameter by transmitting to safe channel easier. Likewise, it is possible to construct the system reinforcing the security with the application of an Identity Based Encryption for transmitting the observed data in a metrological observation environment.

## System model

In this section, we will define the requirements of the system and security protocols of the proposal system and discuss the designing methodology of the system.

### Proposal system

**Fig. 4:**
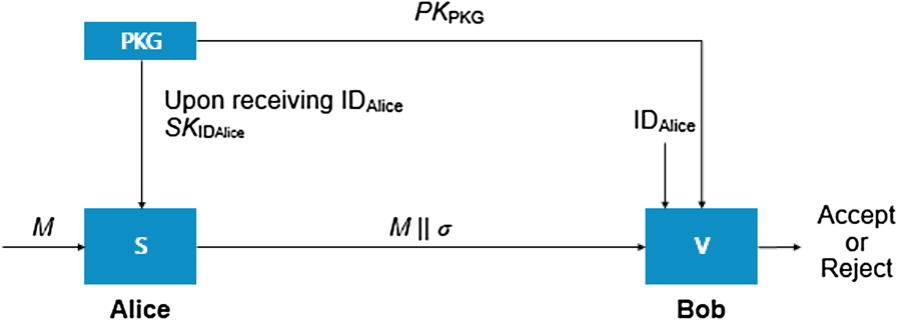
Message transmission using an identity based signature

**Fig. 5:**
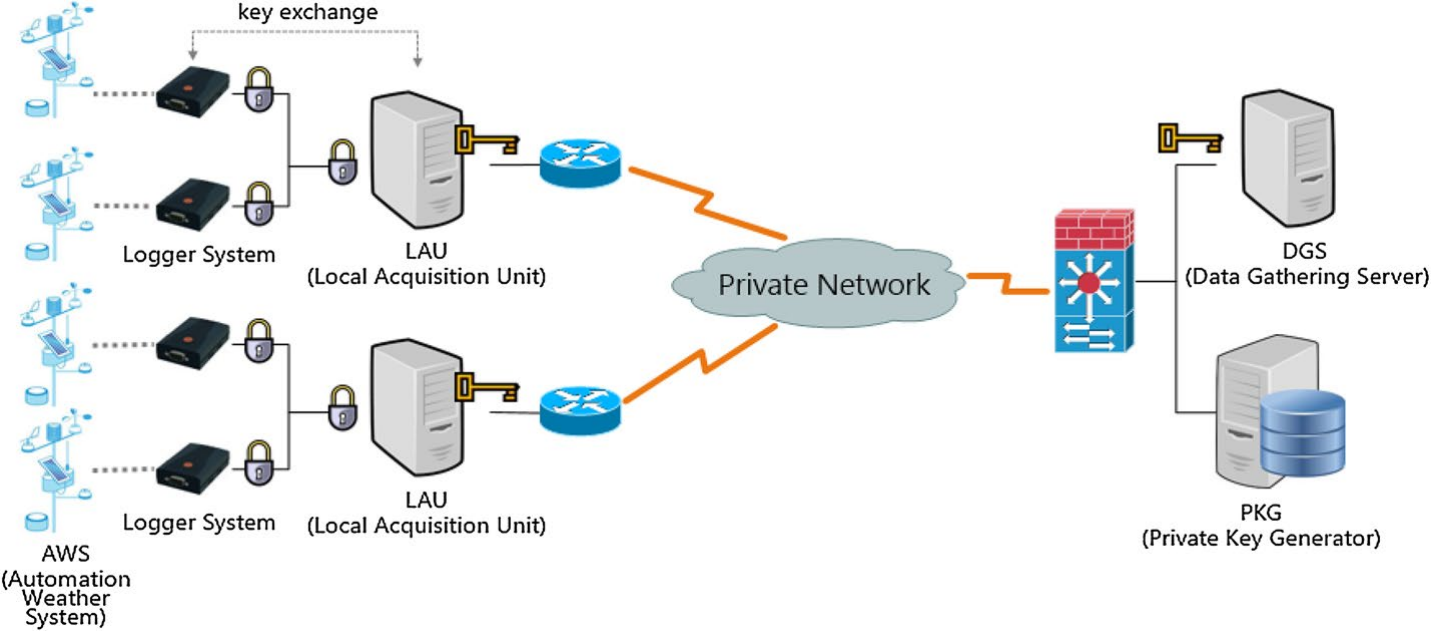
Flowchart for an encrypted communication on weather observation data

Interchanging of meteorological observation data in order to produce weather forecasting needs to consider the integrity factor foremost in the aspect of security. In this paper, an Identity Based Signature was utilized in order to secure the integrity of the meteorological observation data. Figure 4 explains the methodology of message transmission within the application of the proposed system. Figure 5 shows the entire flowchart of the interchange of the meteorological observation system with the application of an Identity Based Signature.

Observation values from the weather sensors get transmitted to the LAU (Local Acquisition Unit) Server after it gets converted into a digital signal through the data logger system. At this point, the data logger system and the LAU Server perform an encrypted communication of symmetry key types using the exchanged security key through the mutual authentication, and the data logger system transmits the observation value to the LAU server every minute. If data cannot be transmitted every minute, either a fault has arisen in the data logger system or an unexpected hijacking has happened. Therefore, an inspection for physical hijacking and responsive actions to the failure need to be performed.

A PKG gets distributed to the LAU server after the generation of private keys with Media Access Control (MAC) information. The LAU server transmits the weather observation values to the Data Gathering Server (DGS) signing it with a private key, and the transmitted signature gets inspected in the DGS. If the transmitted signature from the LAU server coincides with it, then the value is confirmed. If not, then the value is discarded.

### Registration of meteorological observation system and authenticating protocol

In this research, we performed the registration of a new meteorological observation system using the mutual authentication. We utilized the Identity Based Signature to transmit the observation value, and verify the integrity of the transmitting result. In a case where a new weather sensor is added, we differentiated the area performing the mutual authentication between the data logger and the LAU Server from the area transmitting the observed data of the LAU server to the Data Gathering Server (DGS) utilizing the Identity Based Signature.

### Authentication of weather sensor and data logger

In cases where the data logger gets installed, or upgraded to a newer device, we performed a mutual authentication between the data logger and the LAU server. At this moment, if it gets installed at a new observing point, then the LAU server and the data logger interchange the key offline. In addition, the data logger performs an encrypted transmission after it creates the interchanged key with its MAC Address, IP Address, and a generated random number. The LAU server categorizes the MAC addresses and serial numbers of the data logger in order to manage them; generating ${\text{r}}2$ encrypts it to ${\text{E}}_{\text{k}} \left( {{\text{r}}1 \parallel {\text{r}}2} \right)$ with ${\text{r}}1$ to transmit it to the data logger. The data logger decrypts the information from the LAU server and it identifies ${\text{r}}1$ and encrypts ${\text{E}}_{\text{k}} \left( {{\text{r}}2} \right)$ to transmit it to the LAU server. Finally, the LAU server finishes the mutual authentication by performing ${\text{D}}_{\text{k}} ({\text{E}}_{\text{k}} \left( {{\text{r}}2} \right))$ and checking the ${\text{r}}2$ value.

**Fig. 6:**
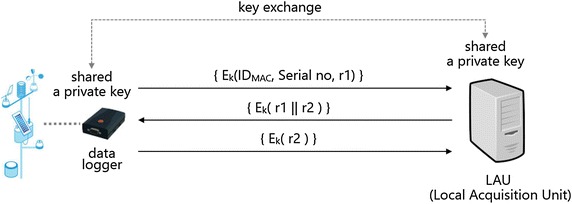
The mutual authentication protocol with the data logger and LAU server

The observation value, measured every minute, gets transmitted to the LAU server through the data logger, and then the LAU server transmits the observation value to the gathering server through the wired or wireless weather communication network. If the data does not get transmitted every minute, then it is a case where faults arose or hijacking occurred by an external attacker, which are necessary to be identified. Also, when the data logger gets replaced due to faults in the device, the procedure shown in Fig. 6 is performed for authentication.

### Authentication and signature of the data logger and data gathering server (DGS)

**Fig. 7:**
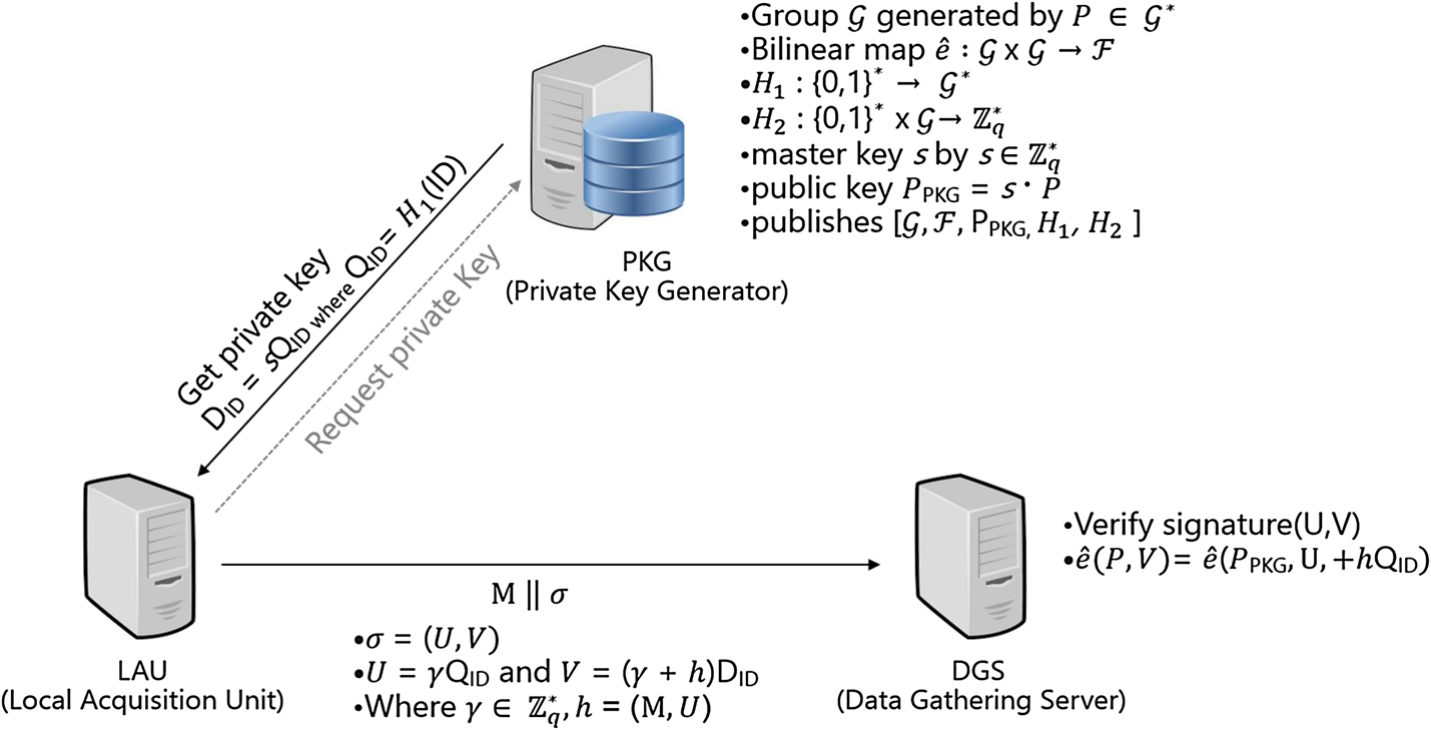
The verification of integrity of weather data using an identity based signature

An important factor about a weather observation value is to verify the integrity that the observed meteorological data has not been forged or falsified and needs to be verified. Figure 7 shows the methodology in order to verify the weather observation value using an Identity Based Signature.

The proposed algorithm was designed based on the Weil pairing IBS and a detailed algorithm is the following (Boneh et al. 2001; Ding and Tsudik 2003).

In the setup stage, the PKG defines group ${\text{g}}$ generated by ${\text{P}} \in {\text{g}}^{ *}$ and ${\hat{\text{e}}}:{\text{g}} \times {\text{g}} \to {\text{F}}$ and two hash functions define ${\text{H}}_{1} :(0, 1)^{ *} \to {\text{g}}^{ *}$ and ${\text{H}}_{2} :(0, 1)^{ *} \times {\text{g}} \to {\text{Z}}_{\text{q}}^{ *}$. The PKG chooses the master key ${\text{s}}$ from ${\text{Z}}_{\text{q}}^{ *}$ and computes the public key ${\text{P}}_{\text{PKG}} = {\text{s}} \times {\text{P}}$. The PKG notifies group ${\text{g}}$ and ${\text{F}}$ and public key ${\text{P}}_{\text{PKG}}$ and hash functions.

The LAU server (signer) requests a private key from the PKG and receives the private key ${\text{D}}_{\text{ID}} = {\text{sQ}}_{\text{ID}}$. The LAU server performs a digital signature of the meteorological observation value by computing ${\text{U}}, {\text{V}}$ and sends them with the observation value to the DGS. At this point, ${\text{U}}$ is ${\text{U}} = {\text{rQ}}_{\text{ID}}$, ${\text{V}}$ is ${\text{V}} = \left( {{\text{r}} + {\text{h}}} \right){\text{D}}_{\text{ID}}$, and ${\text{r}}$ chooses a random number from ${\text{Z}}_{\text{q}}^{ *}$ and ${\text{h}}$ is denoted as ${\text{h}} = {\text{H}}_{2} \left( {{\text{M}}, {\text{U}}} \right)$. The DGS (Data Gathering Server) server (verifier) performs verification by identifying the signature $\left( {{\text{U}}, {\text{V}}} \right)$ of the LAU server by ${\hat{\text{e}}}\left( {{\text{P}},{\text{V}}} \right) = {\hat{\text{e}}}\left( {{\text{U}} + {\text{h}},{\text{P}}_{\text{PKG}} ,{\text{Q}}_{\text{ID}} } \right).$

### Proposed system action

**Fig. 8:**
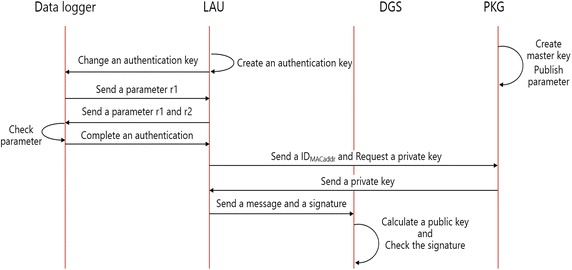
Operating procedure of the proposal system

The LAU server performs a mutual authentication when the data logger is added or replaced. It generates the authentication key and it interchanges the authentication key while installing the data logger. At this point, authentication is performed by a mutual interchange and verification with a random number generated. Observation values collected by the LAU server are transmitted to the Data Gathering Server (DGS) through a dedicated private circuit. The Identity Based Signature is applied to verify the integrity of the observation value transmitted between the LAU and the Data Gathering Server (DGS). The PKG server generates the master key in order to generate the private key with specific information about the LAU server and it releases the private key and the value of the variable. The LAU server signs the observation value with a private key in order to transmit it to the DGS. The DGS identifies the signature of the observation value with a public key to verify its integrity. The operating procedure to authenticate the proposal system is shown in Fig. 8.

## Implementation and comparative analysis

A Weil pairing IBS applied in order for the performance of the proposal system has a low risk of exposure of the key, high performance for the authentication, and no burden into managing the certification. An Identity Based Encryption might seem vulnerable to exposing the key compared to a PKI (Public Key Infrastructure), Safety level is DLP (Discrete Logarithm Problem), which is not different from RSA Algorithm.

An Identity Based Encryption does not leave any information exposed or able to be analyzed by CPA (Chosen Plaintext Attack) and CCA (Chosen Ciphertext Attack). Identity Based Encryption of Weil Pairing ensures IND-ID-CPA which can’t get the ciphertext about the selected plaintext and it ensures IND-ID-CCA which can’t get plaintext information about the selected ciphertext. Additionally, since it is not possible to analogize the ciphertext of a plaintext from an Identity Based Signature, it satisfies the integrity. It also performs a mutual authentication through a digital signature on the message digest, which is a combination of user attributes and messages, which verifies the integrity of message (Naccache 2007; Boneh and Katz 2005; Gentry and Silverberg 2002).

### Implementation

In this paper, the system was set as the following, in order to guarantee the integrity of meteorological observed data and to verify the authenticity of the weather sensors:

A system environment was constructed using an Intel CPU 2.40 GHz (6-Core), 6 GB MEM, 2TB HDD, 32 bit Windows OS and the program language is C++. In order to implement an Identity Based Signature algorithm, a Boneh Lynn Shacham (BLS) Signature module utilizing a PBC (Pairing Based Cryptography) library was used. The PBC library is open-source software implemented with C programming language based on the GMP (GNU Multiple Precision Arithmetic Library) (Crypto.stanford.edu 2016).

The PKG server generates a master key and releases the public parameter to the public. As discussed in 5.2, the LAU server transmits the authentication information to the PKG server in order to generate a private key and it also acquires the private key. To generate a private key, we used a MAC address on a Network Interface Card of the LAU server and the proposed method would ensure the integrity using an Identity Based Signature for the transmission of the observation data from the LAU server to the DGS server. Table 2 shows the test result of the proposed model.

**Table 2:** Result of implementation on proposed model

Category	Result of test
MAC address	C0-3F-D5-XX-XX-XX
Observation data	0,0,0,0,0,-4.7,1.6,1.2,47…
System parameter	628532393961546518621893050439889511258…
Private key	958596650709445949757748689482997019171…
Public key	390263235679716739781014527739492854373…
Message hash	509646011734071305616641800712386292757…
Signature	33644381657097874189819826099858510093…
Compressed	00BB273098C2B9DCF8627520F5137A07D09…

The PKG server creates s system parameter utilizing the MAC address and it calculates a master key in order to generate a private key. Then, using a hash algorithm, it generates a hash value and performs a digital signature. We use a signature and system parameter, message hash, and public key for verification of the generated signature and check the validity by matching the pairing results. The following is the source code for the verification of the signature.

### Processing speed

We evaluated the effectiveness by comparing the Identity Based Signature and the public key- based signature for the transmission of meteorological data. The public key-based signature takes longer for encryption and decryption, authentication, and verification.

In cases where an encryption system was texted with hardware, with that 1000–10,000 × DES) = t(RSA), there is a difference in processing speed, and processing time depending on the encryption system, which was shown as *k*(*DES*) = *k*_*des*_ and *k*(*RSA*) = *k*_*rsa*_. Accordingly, the execution time for encryption is *k*_*rsa*_^*e*^ = 2*k*_*des*_, and the execution time for decryption is *k*_*rsa*_^*d*^ = 2*k*_*des*_. Also, since the authorization processing time was to encrypt a 128 bit digest into a private key, if the encryption processing speed was considered as a constant, it is ${\text{k}}_{\text{rsa}}^{\text{s}} = {\text{k}}_{\text{hash}} + {\text{c}} = {\text{k}}_{\text{des}} + {\text{c}}$, as processing time is equal to authorization, it is ${\text{k}}_{\text{rsa}}^{\text{v}} = {\text{k}}_{\text{des}} + {\text{c}}$ (Choi 2012).

The encryption processing speed of the proposed system model is 11 s and the processing speed of the PKI and DES is shown in Table 3.

**Table 3:** Comparative analysis of the efficiency of the authentication technologies

Category	DES	PKI	Proposed method
Average time	6 ms	12 ms	11 ms

The proposed method provides the same stability with the Public Key Infrastructure structure and processing time for encryption and decryption presents the same time with the symmetry cryptography. It is confirmed that in the process of Setup, Extract, Encrypt and Decrypt when the master key is generated and when utilizing the initial service distributing the system parameter; it takes longer to process time. However, after the initial process is completed, in the process of encryption and decryption, it takes about the same processing time as symmetric cryptography, due to the fact that it utilizes previously saved keys.

### Stability

An Identity Based Encryption is graded safe as a normal RSA. As the DLP (Discrete Logarithm Problem) in the encryption analyzes the safety of cracking keys, it guarantees IND-ID-CPA and IND-ID-CCA, which are preventing attackers from analyzing the targeted ciphertext with achieved information. In addition, authorization of the LAU server is performed by signing the combined digest about system identity and messages. By performing a verifying algorithm through the interchange of an authorization key between the LAU server and the data logger, authorization of the weather sensor can ensure stability from physical attacks.

In order to apply the security authorization protocol in the embedded system, the hardware environment that can run the compiled encryption module needs to be prepared. In the case of a system-based on a sensor network, it is not easy to apply an encryption module due to the miniaturization and low cost. However, the data logger has enough memory and hardware storage space to run and apply a Linux-type embedded operating system, a wired and wireless communication port, a USB port, a TCP communication environment, and an encryption module. Because of this fact, unlike the previous mini-communication systems, in a meteorological observation data-transmitting environment, it is possible to apply an Identification-Based Encryption protocol. Table 4 shows the comparative analysis of the stability depending on the communication technology.

**Table 4:** Comparative analysis of the stability of the communication method

Category	USN/M2M	PKI	Proposed method
Integrity of data	Limited	Enabled	Enabled
Reply attack	Limited	Disabled	Disabled
Verification of quality of data	Disabled	Disabled	Enabled
Physical attack	Weak	Weak	Strong
Convenience of implementation	Easy	Difficult	Middle

The proposed system model guarantees the integrity of the observation data. Also, it provides the stability against Man in the Middle Attacks and Reply Attacks and it causes difficulties for external attackers to perform physical attacks by performing a mutual authentication of the weather sensors of the terminal. As a result, the Identity Based Encryption provides more convenience in implementation than PKI.

## Conclusion

Weather information is important information to most people regardless of their occupation, gender, or age; it is even directly related to the even national economy and security. It is a core consideration for the flight, landing, and taking off of fighter jets, and for warships, and it plays more an important role in the information of strategy, tactics, and trend information. Considering the security of weather information, insecure weather information could be a huge threat to national security and the economy. It is essential, not optional.

In cases where the PKI is applied in order to build an encrypted communication environment for the safe transmission of meteorological observation data, efficient verification procedures of separated authentication management servers and issued authentication are required. Therefore, if the PKI is applied for the safe transmission of meteorological observation data, there may be difficulties in the implementation of operating environments where communication/computation load-lightening is required.

Thus, in this paper, the IBS (Identity Based Signature) was applied for the safe transmission of weather data, where integrity and confidentiality were achieved as a discernment factor by generating a private key.

As a result, in order to satisfy the core factors of information security in meteorological observation systems of the Internet of Things, applying the IBE and IBS provides the most efficient and stable performance.
